# Draft Genome Sequences of Fungus *Aspergillus calidoustus*

**DOI:** 10.1128/genomeA.00102-16

**Published:** 2016-03-10

**Authors:** Fabian Horn, Jörg Linde, Derek J. Mattern, Grit Walther, Reinhard Guthke, Kirstin Scherlach, Karin Martin, Axel A. Brakhage, Lutz Petzke, Vito Valiante

**Affiliations:** a Department of Systems Biology/Bioinformatics, Leibniz Institute for Natural Product Research and Infection Biology, Hans Knöll Institute (HKI), Jena, Germany; b Department of Molecular and Applied Microbiology, HKI, Jena, Germany; c Institute for Microbiology, Friedrich Schiller University, Jena, Germany; d National Reference Center for Invasive Fungal Infections, HKI, Jena, Germany; e Department of Biomolecular Chemistry, HKI, Jena, Germany; f Bio Pilot Plant, HKI, Jena, Germany; g BASF SE, Ludwigshafen, Germany; h Leibniz Junior Research Group-Biobricks of Microbial Natural Product Syntheses, HKI, Jena, Germany

## Abstract

Here, we report the draft genome sequence of *Aspergillus calidoustus* (strain SF006504)*.* The functional annotation of *A. calidoustus* predicts a relatively large number of secondary metabolite gene clusters. The presented genome sequence builds the basis for further genome mining.

## GENOME ANNOUNCEMENT

*Aspergillus* is the most studied filamentous genus in the ascomycetes division. It contains well-known human pathogens (e.g., *A. fumigatus*, *A. terreus*), fermentation agents of Asian food (e.g., *A. oryzae*), and different industrial producers (e.g., *A. niger* and *A. flavus*) (1). Additionally, the genus is well studied because of its immense source of natural products (2). The worldwide distributed *Aspergillus calidoustus* was recently separated from the mesophilic species *A. ustus* (3). *A. calidoustus* was predominantly isolated from indoor environments and immunocompromised patients (3, 4).

Strain SF006504 was identified as *A. calidoustus* based on the β-tubulin sequence (100% similarity with the ex-type strain CBS 121601). Genomic DNA was obtained from a sample cultured in *Aspergillus* minimal medium (AMM) (5). For Illumina MiSeq V2 DNA sequencing, four different libraries were prepared (paired-end [PE], 2kbp-MP, 5kbp-MP, 8kbp-MP). The genome assembly was generated using Allpaths-LG (6). RNA-seq data was obtained for three replicates for standard growth, hypoxic, and iron depletion conditions. Sequencing was performed with Illumina HiSeq 2000 (LGC Genomics, Berlin).

For gene prediction, we customized and augmented the pipeline of Haas et al. (7) as previously described (8), applying *ab initio* prediction tools, tools incorporating transcriptome data, and protein alignments. If applicable, the parameter sets for each tool were trained using the transcriptome assembly. All gene predictions were combined using EVidenceModeller and PASA (9).

For *ab initio* gene prediction, we applied GeneMark-ES (10), Augustus (11), and SNAP (12). Transcriptome data was incorporated into Augustus (11), FGENESH (13), and PASA (14), which utilized genome-guided and genome-independent transcriptome assemblies produced by Trinity (15) and Cufflinks (16).

Protein alignments were obtained by mapping protein sequences from *A. fumigatus, A. nidulans, A. oryzae, A. terreus*, and *A. niger* (downloaded from the Aspergillus Genome Database [17]), using Exonerate (18) and Scipio (19).

Functional annotation was performed using Blast2GO (20) and InterproScan (21). Gene descriptions were obtained by blasting the predicted protein sequences against fungal UniProt KnowledgeBase. Matches with e-values below 10^−5^, 70% sequence identity, and a subject hit length of 70% were considered as highly similar. Secondary metabolite gene clusters were predicted using SMURF (22).

DNA-sequencing resulted in 59,066,664 raw reads, where 50,376,036 reads passed our quality-filter (estimated genome coverage, 300-fold) and have been used for genome assembly. The resulting assembly consists of 78 scaffolds and 41.1 Mbp (*N*_50_ 3.2Mbp; *N*_90_ 493 kbp). The total G+C content was 51%. RNA-sequencing resulted in a total of 393,543,839 raw reads and 352,698,587 preprocessed reads (estimated genome coverage, 850-fold). The final structural gene prediction resulted in 15,139 gene models and 15,537 transcripts. 484 eukaryotic core proteins were identified using CEGMA (23). The coding density of the genome was 60%. We assigned functional names to 5,572 transcripts, gene ontology (GO) categories to 8,352 transcripts, and protein domains to 13,610 transcripts. 3,771 transcripts were predicted to contain transmembrane domains, and 749 transcripts have been assigned to 53 secondary metabolite gene clusters. GO annotations have been made available for downstream analysis at FungiFun2 (24).

### Nucleotide sequence accession numbers.

This genome project was uploaded to DDBJ/ENA/GenBank and is available under accession numbers CDMC01000001 to CDMC01000078. This paper describes the first version of the genome. Genome data and additional information are also available at the HKI Genome Resource (http://www.genome-resource.de/).
